# The role of community pharmacists in medicines optimisation for housebound people: A scoping review

**DOI:** 10.1371/journal.pone.0331294

**Published:** 2025-09-11

**Authors:** Jennifer Blease, Yahvi Bhonsle, Rose Ireson, Albert Farroha, Greg Westley, Richard Cooper, Daniel Hind

**Affiliations:** 1 The Medical School, University of Sheffield, Sheffield, United Kingdom; 2 NHS South Yorkshire ICB, South Yorkshire, United Kingdom; 3 School of Medicine and Population Health, University of Sheffield, United Kingdom; 4 School of Healthcare, Level 10, Worsley Building, University of Leeds, Leeds, United Kingdom; Group for Technical Assistance / Asian College for Advance Studies, Purbanchal University, NEPAL

## Abstract

**Background:**

An increasingly ageing population presents many challenges for healthcare systems, including how to support older adults who are more likely to be both housebound and have complex medication needs. Community pharmacists may play a key role in medicines optimisation for this vulnerable population, however, the extent of literature exploring this topic is unclear.

**Objective:**

To map existing literature on the role of community pharmacists in medicines optimisation for housebound older adults in the United Kingdom (UK).

**Methods:**

A scoping review was conducted following PRISMA-ScR guidelines. Peer-reviewed primary research and grey literature published since 2000 were searched using relevant databases and websites. Data was charted using a standardised form based on TIDieR guidelines and EPOC taxonomies. A narrative synthesis was conducted to summarise and interpret the findings from included studies.

**Results:**

Seven sources were included in the review – five peer-reviewed articles and two grey literature reports. Interventions consisted of domiciliary medication reviews conducted by pharmacists. Key medicines optimisation strategies addressed were medication review, deprescribing, addressing polypharmacy and facilitating communication between providers. Reported outcomes included identification of widespread issues with polypharmacy and medication-related problems, reduced hospital admissions, cost savings and improved patient care. Gaps identified were limited generalisability, lack of comparisons to standard care, and under-representation of minority groups.

**Conclusions:**

The literature indicates promise for the role of community pharmacists in medicines optimisation for housebound older adults through domiciliary services. However, more research is needed to evaluate the effectiveness and feasibility of pharmacist-led interventions in this setting. Addressing identified gaps will help inform pharmacists’ roles in supporting medication needs of housebound patients.

## Introduction

### Rationale

The number of older adults in England and Wales is increasing rapidly, with the population aged 65 and over growing by over 1.8 million between 2011 and 2021 [1]. This ageing population presents significant healthcare challenges, as older individuals often suffer from multiple chronic conditions and take numerous medications (polypharmacy). Over 10% of those aged 65 and above take at least 8 different prescribed medications each week [2,3]. Polypharmacy increases the risks of drug interactions, impaired medication adherence, reduced quality of life [2,4–6], and adverse drug reactions, which are a leading cause of hospital admissions [4,5]. A person taking ten or more medications is 300% more likely to be admitted to hospital due to adverse drug reactions [7]. Furthermore, around 6.5% of hospital admissions are caused by adverse effects of medicines, rising to 20% in the over 65 age group, with two-thirds considered preventable [7].

An important, but ill-defined, target population for hospital admissions are housebound older adults. Housebound (US: ‘Homebound’) is generally defined as the condition in which a community-dwelling adult is confined to the home without support, implying a need for help with activities of daily living, mobility limitation and frailty [8,9]. Unlike care home residents, who – in the UK – benefit from regular multidisciplinary reviews and prioritisation for medication review [10] — housebound older adults face challenges with medication management due to isolation and reduced healthcare access [11,12]. The Community Pharmacy Contractual Framework focuses the currently shrinking workforce on in-pharmacy services, such as blood pressure monitoring and new medicine counselling, with no infrastructure for home visits, effectively excluding those unable to attend [13]. This creates a disparity in pharmaceutical care access for housebound older adults compared to care home residents, despite similar polypharmacy risks. Housebound older adults are missing services guaranteed to care home residents: weekly multidisciplinary team rounds, proactive personalised care planning within 7 days of health changes, systematic medication reviews , structured information sharing protocols between providers, and regular clinical oversight from a named healthcare lead.

Evidence syntheses have highlighted the need for further research into structured medication reviews for housebound older adults [14] and have called for evaluations of community pharmacist-led home visit models [15]. Some reviews also advocate for integrated approaches that offer proactive medicines optimisation comparable in quality and scope to those provided in care home services [14,15]. However, care home services are typically delivered through GP practices and practice-based pharmacists. In contrast, community pharmacy-led home visits is an approach that remains underexplored but may offer enhanced accessibility, continuity, and a broader reach beyond existing GP-led models. Recent studies by Latif et al. [16] and Kayyali et al. [17] found that pharmacist-led domiciliary medication reviews (dMURs) could identify and address medication-related problems, potentially preventing hospital admissions. However, these initiatives fall short of the comprehensive medicines optimisation approach proposed by the National Institute of Health and Care Excellence (NICE) in England [18].

Community pharmacists are well-positioned to support medicines optimisation for older adults [2,6]. The National Health Service (NHS) Long Term Plan commits to expanding access to medicines reviews and integrating pharmacists into local health teams [2,6]. However, a robust system for supporting the wider population of housebound older adults is lacking.

Despite clear policy recognition of this disparity, no comprehensive synthesis exists examining how community pharmacists contribute to medicines optimisation for housebound older adults. Previous reviews have not examined the full spectrum of medicines optimisation activities beyond basic medication reviews. This represents a knowledge gap given the UK’s unique healthcare structure and recent policy developments around structured medication reviews. This review provides the first systematic mapping of community pharmacist involvement in medicines optimisation specifically for housebound older adults within the UK healthcare context, examining both published research and grey literature to identify priority areas for future service development and research.

### Aims and objectives

The primary research question is: What roles do community pharmacists currently play in medicines optimisation for housebound older adults in the UK, and what gaps exist in current service provision and research evidence?

This scoping review aimed to address the knowledge gap surrounding the role of community pharmacists in medicines optimisation for housebound older adults in the UK. By systematically mapping the existing literature, it will:

1. Map the existing evidence on the roles currently undertaken by community pharmacists in supporting medicines optimisation for housebound older adults.
2. Identify examples of pharmacist-led services that extend beyond medication reviews to more holistic medicines optimisation practices.
3. Examine evidence of collaboration or integration between community pharmacy and health and social care services in the delivery of medicines-related care.
4. Determine gaps in the literature and outline priorities for future research and service development.

## Methods

### Protocol and registration

The protocol (S1 Protocol) was drafted by two reviewers using the PRISMA Extension for Scoping Reviews and subsequently registered with the Open Science Framework. It was then published on ORDA [19]. This scoping review is reported in accordance with the “Preferred Reporting Items for Systematic reviews and Meta-Analyses extension for Scoping Reviews (PRISMA-ScR) Checklist” [20] (S2 Checklist).

### Eligibility criteria

The eligibility criteria were formed with a specific view to determining the scope of literature regarding pharmaceutical services for housebound people, and therefore any reports concerning other home-based interventions for participants who could freely leave their homes were not included. Reports investigating any type of pharmacist intervention, including medication reviews, were included if they fit all other criteria. Peer reviewed primary research was accepted if it was published after 2000; was undertaken in the United Kingdom; and published in English. Evidence suggests that the exclusion of non-English studies rarely affects effect estimates or review conclusions [21]. Any reports including patients in care homes, hospitals, or non-domiciliary settings were excluded. Care home residents were excluded from this review as, in the UK, they receive structured pharmaceutical care through the Enhanced Health in Care Homes framework, which promotes weekly pharmacy-led medication reviews as best practice and established medicines optimisation protocols, whereas housebound community-dwelling older adults lack access to these systematic pharmacy services despite having similar polypharmacy risks and medication management needs [10].

A full copy of the inclusion and exclusion criteria for this review is provided in the appendices (S3 Appendix).

### Information sources and search strategy

The search strategy is listed in its entirety in the appendices (S4 Appendix). Searches were last completed 22^nd^ September 2024 on MEDLINE and EMBASE. Best evidence suggests that MEDLINE and EMBASE would capture the most relevant studies with little impact on results from missing studies [22–24]. Information from the articles found from the search were transferred onto Rayyan for review.

### Selection of sources of evidence

Rayyan was used to aid the selection of sources of evidence, and a PRISMA flow diagram developed to demonstrate the process. The titles and abstracts from articles identified were screened independently by five reviewers to increase consistency, with any conflicts resolved through discussion. Meta-epidemiological research shows that single screening is suboptimal [25], especially with inexperienced reviewers [26]. Full texts were located for the remaining reports, and these underwent a secondary screening and data charting process.

The list of identified websites was searched for grey literature (S5 Appendix).

Search terms included the site name, ‘housebound’, ‘pharmacy/ist/eutical’, ‘domiciliary’, and ‘medication review’.

### Data charting process

Data was charted on a standardised form developed by two reviewers using a small sample of eligible papers. The final form had 45 carefully chosen variables to extract the most amount of information from the texts. Four reviewers independently charted the data, with any conflicts resolved through discussion. Where needed, authors were contacted for information not available in the publication. We did not assess study quality or risk of bias, consistent with current methodological guidance that scoping reviews do not require critical appraisal [27–29].

### Data items

A comprehensive list of the data charted for is included in the appendices (S6 Appendix). Within these variables, reviewers reported on characteristics of the literature, participant characteristics, details of the intervention elements, barriers and facilitators to engagement, and further utilisation or research recommendations. The sources were also compared with a list of interventions, services or initiatives related to medicines optimisation, included in the protocol.

### Synthesis of results

A narrative synthesis was undertaken, to provide a structured summary of both the included papers and grey literature. We extracted descriptive data using a standardised data charting form based on TIDieR guidelines and EPOC taxonomies. Four reviewers (JB, YB, RI, AF) independently charted the data, capturing key intervention characteristics, objectives, implementation features, and reported outcomes. The charted data were then analysed using an iterative narrative synthesis approach. This process involved identifying patterns and relationships across studies, grouping similar intervention components and contextual factors, and organising findings into coherent thematic categories aligned with the frameworks used. Discrepancies in data interpretation were resolved through discussion among reviewers. Senior members of the research team (RC, DH) provided oversight and methodological guidance during synthesis, ensuring consistency and rigour in the identification of cross-cutting themes and gaps in the literature. This approach allowed for identification of patterns and relationships across studies, while accommodating variations in study design and outcomes. The synthesis aimed to highlight common themes, contextual factors, and areas of divergence relevant to the review.

## Results

### Selection of sources of evidence

The search initially identified 195 articles. Duplicates were then identified and removed, leaving 188 potentially eligible records. Screening at the title and abstract stage excluded 178 records as irrelevant. The full texts for all 10 articles were retrieved.

**Fig 1:**
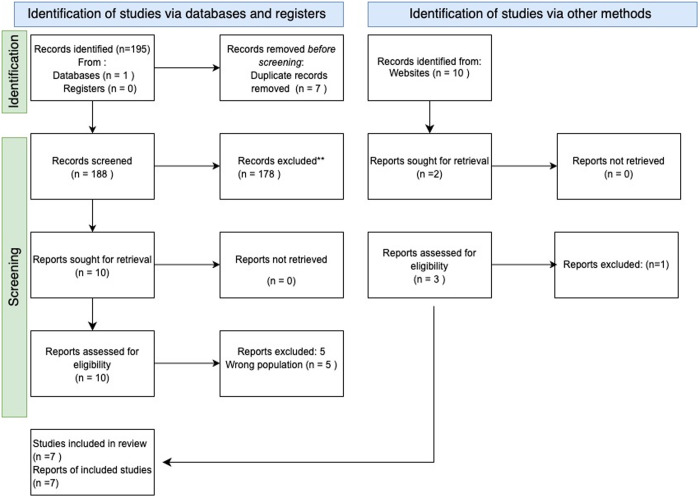
PRISMA 2020 flowchart diagram.

Sources were excluded at the full-text level due to ineligible population (n = 5) [30–34]. These had undertaken research into delivery of a similar service for people who were not housebound, or who had been admitted into care homes. This secondary screening left five eligible reports for the review. This process was reported in a PRISMA diagram (Fig 1).

The search process for grey literature found 3 articles, of which two were eligible. All seven sources were data charted to develop a detailed understanding.

### Study characteristics

Data sources consisted of peer reviewed articles (n = 3) [16,17,35] and conference abstracts (n = 2) [36,37] between 2017 and 2022 (Table 1), including cross sectional studies (n = 3) [16,17,37], and case series (n = 2) [35,36]. Amongst the two grey literature sources there was one report [38] and one online publication regarding guidance recommendations [39], both published by the Royal Pharmaceutical Society.

**Table 1:** Study characteristics.

Author	Document type (grey literature; peer reviewed article; conference abstract)	Study design	Patient group	Numbers and types of medicines	Brief name
Kayyali R, Funnell G, Harrap N, Patel A. 2018	Peer reviewed article	Cross-sectional study	133 patients – 49–98, housebound, mean age 81.7 years	Average 9.4 medications	dMUR
Garfield SF, Wheeler C, Etkind M, Ogunleye D, Williams M, Boucher C, et al. 2022	Conference abstract	Cross-sectional study	50 patients – mean age 68 years (26–93 years)	1 long term medication	Medication safety interview
Hurley D. 2018	Conference abstract	Case series	69 patients – mean age 81.2 + /- 8.1 years 90% of patients housebound – patients had been referred to falls service	Not stated	Full level 3 medication review
Latif A, Mandane B, Anderson E, Barraclough C, Travis S. 2018	Peer reveiwed article	Cross-sectional study	1092 – patients were housebound, 76.9% were over 75 years old	Not stated	dMUR
Souter C, Kinnear A, Kinnear M, Mead G. 2017	Peer reviewed article	Case series	35 patients completed the study- 18 inpatients, and 17 outpatients mean age 74.2	Not explicitly stated	Pharmacist complex intervention
Royal Pharmaceutical society 2014	Grey literature	N/a	322 housebound patients	Not stated	dMUR
Royal Pharmaceutical society	Grey literature	N/a	169 frail elderly housebound patients across 7 GP practices	Not stated	Multidisciplinary pharmacy review

Patient population sizes ranged from 35 [35] to 1092 [16]. The terminology and wording of the interventions delivered to patients varied: three studies referred to domiciliary medicines use reviews [16,17,38] but elsewhere, pharmacist complex intervention [35], full level 3 medication review [36] and multidisciplinary review [39] were used to describe interventions with. One study not specifically naming the intervention [37]. Five sources explicitly described patient demographics; most had a female majority (n = 4) [16,17,35,37]. Heterogeneity in patient populations limited comparability across studies. Latif included a broad housebound population [16]; Kayyali focused on older adults with complex social needs [17]; Hurley on frail, fall-prone patients [36]; Souter on post-stroke patients [35]; and Garfield included younger adults (mean age 68) with varied conditions [37]. Differences in clinical needs were marked: Souter involved patients requiring stroke rehabilitation [35]; Hurley targeted those at risk of falls [36]; Latif encompassed a wide range of conditions, diluting condition-specific insights [16]. Age and frailty also varied—Garfield had a younger cohort [37] compared to Kayyali and Hurley, both with mean ages over 81—limiting generalisability to older populations [17,36]. Levels of complexity and dependency were uneven: Kayyali described substantial social care and functional needs [17], while Garfield lacked detail on patient dependency [37]. Definitions of “housebound” varied. Latif defined patients as “unable to attend the pharmacy” and not in residential or nursing care [16]. Kayyali included those “who would otherwise not be able to access the pharmacy,” often referred by GPs or district nurses [17]. Souter did not use the term but included patients discharged home post-stroke, implying housebound status through functional limitation and exclusion of those in long-term care [35]. Garfield referred to people “unable to attend pharmacies,” but did not specify criteria [37]. Hurley did not define housebound [36].

### Intervention objectives

Study interventions involved the use of medication reviews (n = 6) [16,17,35,36,38,39]; patient education and counselling (n = 4) [16,17,37,38]; deprescribing (n = 4) [16,36,38,39]; adherence support (n = 3) [16,17,35]; addressing polypharmacy and inappropriate prescribing (n = 6) [16,17,35,36,38,39]; facilitating communication between healthcare providers (n = 5) [16,17,35,37–39]; and use of technology or information sharing to support medicines optimisation (n = 2) [16,39] (Table 2).

**Table 2:** Intervention objectives.

Author	Medication review	Patient education and counselling	Deprescribing	Adherence support	Addressing polypharmacy and inappropriate prescribing	Facilitating communication between healthcare providers	Use of technology or information sharing to support medicines optimisation	Rationale, evidence-base theory or goal of intervention elements	EPOC taxonomy Intervention category:
Kayyali R, Funnell G, Harrap N, Patel A. 2018	Yes	Yes	No	Yes	Yes	Yes	No	Medication support and social care needs of housebound older patients	Site of service delivery
Garfield SF, Wheeler C, Etkind M, Ogunleye D, Williams M, Boucher C, et al. 2022	No	Yes	No	No	No	Yes	No	Safe medicine practices for housebound patients	Use of information and communication technology and educational materials
Hurley D. 2018	Yes	No	Yes	No	Yes	No	No	Reduce hospital admissions for patients at risk of falls	Site of service delivery
Latif A, Mandane B, Anderson E, Barraclough C, Travis S. 2018	Yes	Yes	Yes	Yes	Yes	Yes	Yes	To evaluate feasibility of providing dMURs for housebound patients	Site of service delivery
Souter C, Kinnear A, Kinnear M, Mead G. 2017	Yes	No	No	Yes	Yes	Yes	No	Testing practicality, acceptability, and feasibility to inform the design of a definitive RCT	Site of service delivery
Royal Pharmaceutical society 2014	Yes	Yes	Yes	No	Yes	No	No	Not stated	Site of service delivery

Five of the data sources described a specific intervention and specified the goals and rationale [16,17,35,37,38] (Table 2). The goal of intervention elements included: evaluating medication needs (n = 4) [16,17,37,38]; feasibility tests (n = 2) [16,35]; an exploration of medicines practices and safety (n = 1) [37]; and an assessment of whether the intervention would reduce hospital admissions (n = 1) [38]. Rationale for all five interventions centred around barriers faced by the ageing population in accessing healthcare services and effectively using medicines, especially as they have a higher risk of likelihood of multiple comorbidities and therefore polypharmacy. EPOC taxonomy intervention categories were site of service delivery (n = 6) [16,17,35,36,38,39], and use of information and communication technology and Educational materials (n = 1) [37].

### Intervention characteristics

Where stated, physical materials used included the PharmOutcomes system (n = 2) [16,35], and the dMUR form (n = 1) [17] (Table 3). One source specified an intervention cost of a £56 reimbursement alongside the standard MUR payment of £28 per dMUR carried out by the pharmacist [16].

**Table 3:** Intervention characteristics.

Author	Physical or informational materials used (information materials; training materials)	Intervention costs/resource requirements	Intervention duration	Intervention provider background/expertise (pharmacist; nurse; physician; multidisciplinary team)	Number of intervention providers	Any specific training given to providers	Organisation: Type, size, number of study sites, ratings	Location: Area demographics	Necessary infrastructure or relevant features of locations
Kayyali R, Funnell G, Harrap N, Patel A. 2018	dMUR form	Cost not stated	Each visit was completed within 30–45 min	Community pharmacist following MUR training	12 pharmacists	dMUR information sessions	133 patients, 12 community pharmacists	London Borough of Richmond, UK	Community pharmacist to conduct review in home of patient
Garfield SF, Wheeler C, Etkind M, Ogunleye D, Williams M, Boucher C, et al. 2022	Not explicitly stated	Cost not stated	Not stated	Pharmacist	Not stated	None sated	50 people interviewed remotely	Uk wide	Not stated
Hurley D. 2018	GP records system	Cost of intervention not stated	Not stated	Pharmacist	Not stated	Not stated	Not stated	East Staffordshire, UK	Device to review patient records
Latif A, Mandane B, Anderson E, Barraclough C, Travis S. 2018	PharmOutcomes system	£56 reimbursement fee for each dMUR on top of the standard £28 MUR fee	Not stated	Community pharmacists	91 pharmacies	Interested pharmacies were provided a service specification& a checklist	91 pharmacies undertaking 1092 reviews	Nottinghamshire and Derbyshire	Community Pharmacist
Souter C, Kinnear A, Kinnear M, Mead G. 2017	Machine for BP measurements, GP patient record systems,	Not stated	Not stated	Community pharmacists	Not stated	Not stated	Not stated	Not stated	Community pharmacist to perform review
Royal Pharmaceutical society 2014	Not stated	Not stated	Not stated	Pharmacist in primary care	Not stated	Not stated	Not stated	Croydon	Not stated
Royal Pharmaceutical society	Not stated	Not stated	Not stated	Not stated	Not stated	Not stated	7 GP practices	Scotland	Not stated

Five studies used a pharmacist to conduct the intervention [16,17,35,36,38]; one used a researcher [37] and one did not state the professional [39] (Table 3). The sources were not clear on the number of intervention providers, one stated the use of one pharmacist [35], another stated the use of twelve pharmacists [17], one stated the involvement of 91 pharmacies [16] and four did not specify [16,36–39]. One source gave official training to the pharmacists [17], whereas one provided guidance to the pharmacists completing the reviews [16].

Intervention locations varied: one referred to the United Kingdom more generally [37] but most were related to specific areas including Nottinghamshire and Derbyshire (n = 1) [16], London (n = 1) [17], East Staffordshire (n = 1) [36] Croydon (n = 1) [38], and Scotland (n = 1) [39]. Two sources did not specify locations [35,36]. One source stated the duration of each review, between 30–45 minutes [17].

### Intervention delivery

Two sources referred to initial contact with the patient and/or a carer to organise a suitable time for a meeting, done either by a pharmacist [17], or unspecified [36] (Table 4). Most interventions were delivered face-to-face (n = 5) [16,17,35,36,38]; one was not stated [39], and one was delivered over video or telephone calls [37]. All were delivered on an individual basis except one, which did not specify [37]. Similarly, all were delivered in patients homes except two, which did not specify [37,38]. Follow-up appointments were conducted in two sources [35,36]. Visit frequency varied substantially. Latif involved a single opportunistic dMUR per patient, with low average numbers per pharmacy, reflecting limited capacity and non-mandated follow-up [16]. Kayyali delivered a mean of 2.4 visits, shaped by proactive identification, GP collaboration, and broader optimisation goals [17]. Souter scheduled three visits per patient as part of a protocolised trial [35]. Frequency in Garfield and Hurley was unclear or fixed at one; both lacked detail on service design or rationale, limiting interpretation [36,37].

**Table 4:** Intervention delivery.

Author	Procedures, activities, processes used	Modes of delivery (e.g. Face-to-face; telephone; Internet)	Whether delivered individually or in a group	Number of locations	Number of times delivered	Schedule of delivery	If intervention was personalised or adapted, how and why (e.g. based on patient characteristics)	- If/how adherence or fidelity was assessed (e.g. independent assessors; validated tools)
Kayyali R, Funnell G, Harrap N, Patel A. 2018	Telephone call to prospective patients to organise visit with family/carer. Review completed and data analysed	Initial phone call to organise a suitable time for subsequent home visit	Individually	Patients home	One dMUR per patient	Visits took place between May 2015 and January 2016	The pharmacists used the same form for each patient. The exact intervention would depend on the medication prescribed and the individual’s circumstances	Pharmacist service lead assessed forms for completeness and guidance when necessary
Garfield SF, Wheeler C, Etkind M, Ogunleye D, Williams M, Boucher C, et al. 2022	Participants recruited through personal contact or social media, interviews conducted and data analysis carried out.	Telephone or video conference	Individually	Non stated – interviews were remote	Once per patient	Not stated	initial delivery the same, however intervention dependent on patient medication. only medications with no indication or limited efficacy, potentially contributing to falls, were deprescribed.	Not stated
Hurley D. 2018	Medical records reviewed of patients referred to falls service, meeting arranged and carried out, changed were made at GP surgery and followed up after 1–2 months	Medical records reviewed, face to face meeting with patient	Individually	Not stated	One review per patient – follow up after 1–2 months	None stated	None Stated	Not stated
Latif A, Mandane B, Anderson E, Barraclough C, Travis S. 2018	Service advertised to pharmacies and those expressing interest given information. Review conducted and risk of hospital admission scored. Results recorded on PharmOutcomes.	Face to face visit	Individually	Patients homes	Once per patient	April 16 to march 17	The delivery was the same for each patient, but the exact intervention and outcome would vary dependent on the individual’s circumstances.	Not stated
Souter C, Kinnear A, Kinnear M, Mead G. 2017	Patients recruited following stroke unit discharge and permission obtained. Interviews carried out at 1, 3 and 6 months – concerns highlighted with GP	Face to face visit	Individually	Patients homes	3	Visit at 1, 3 and 6 months	Initial delivery the same, but specific actions taken dependent on patient’s circumstances	Not stated
Royal Pharmaceutical society 2014	Not stated	Face to face	Individually	Patients homes	Not stated	Not stated	None Stated	Not stated
Royal Pharmaceutical society	Not stated	Face to face	Individually	Patients homes	Not stated	Not stated	None Stated	Not stated

Four intervention deliveries were personalised according to the patients’ situations (n = 4) [16,17,35,36]. Assessment of fidelity was only mentioned once in which dMUR forms were assessed for completeness by pharmacist service lead [17].

Intervention duration lasted either 6 months (n = 2) [35,38], 9 months (n = 1) [17], or 12 months (n = 1) [16].

TIDieR item 10 has not been reported as no modifications were reported by any of the included papers.

### Findings

There was variation in the extent of pharmacist activity across the interventions and sources: four referred to only one instance of a pharmacist activity per patient [16,17,36,37], with another referring to one additional follow-up per patient [36]. In one study, three pharmacist visits per patient were reported [35] (Table 5). Outcome Characteristics included medication access issues (n = 3) [17,35,37], risk of hospital admissions (n = 3) [36,38,39], polypharmacy (n = 2) [17,38], patient- reported concerns (n = 3) [35,37,40], adherence (n = 4) [17,35,37,40] side effects (n = 4) [17,35,36,40], and prescribing appropriateness (n = 6) [16,17,35–38]. One paper described experiences of twelve pharmacists following testimonies from those involved [16], and all sources reported that the dMUR highlighted issues with medications.

**Table 5:** Intervention findings.

Author	Number of instances of pharmacist activity	Outcome characteristics	Experiences of pharmacists, patients and other stakeholders	Outcomes in relation to medicines optimisation	Prescribing appropriateness (e.g., number and type of medication changes, use of tools like MAI, Beers criteria)	Adverse drug reactions	Adherence
Kayyali R, Funnell G, Harrap N, Patel A. 2018	One visit per patient	Issues with medication access, clinical issues, cognitive issues, social care issues, medication for disposal, polypharmacy, adherence	Not stated	Not explicitly stated, 52 patients with medication for disposal, 13 patients who no longer need medication, 14 taking medication the wrong way	13 cases of patients taking meds they no longer needed	37 cases of adverse effects due to drugs	14 cases of patients taking medication the wrong way
Garfield SF, Wheeler C, Etkind M, Ogunleye D, Williams M, Boucher C, et al. 2022	One per patient	Medicine safety issues of omitting doses, and use of less effective formulations, issues obtaining medications	Not stated	Not stated	Some reports of less effective formulations being used	None stated	Not stated
Hurley D. 2018	One visit and one follow up per patient	Medications with no indication or limited efficacy potentially contributing to falls	Not stated	124 medications deprescribed in 64 patients – 38 of these considered as contributing to poor balance in 38 patients	124 medications deprescribed in 64 patients	38 drugs considered to contribute to falls risk	Not stated
Latif A, Mandane B, Anderson E, Barraclough C, Travis S. 2018	Once per patient	Patient reported concerns, reported side effects, reported missed doses, identification of discontinued medications, security of drugs, stockpiling medications, expired medications	12 pharmacist testimonies were taken and all stated that the dmur highlighted issues with patients’ medications	Two thirds of patients reported concerns, missed doses or side effects over 1/4 had expired or stockpiled medications	58 change of dose forms recommended	77 cases likely to prevent emergency hospital admission – 178 cases of reported side effect	Not stated
Souter C, Kinnear A, Kinnear M, Mead G. 2017	3 visits	Bp and cholesterol measurements, lifestyle records, social and practical support, mmse and current medications (effects and physical abilities of device use.	Not stated	104 care issues identified, (mean 5.8 per patient) including additional, unnecessary and wrong medicines and adverse reactions, interactions or inappropriate compliance.	Additional, unnecessary, wrong and doses too low	11 cases	18 cases of inappropriate compliance
Royal Pharmaceutical society 2014	Not stated	Polypharmacy and risk of hospital admissions	Not stated	83 emergency hospital admissions prevented	Not stated	Not stated	Not stated
Royal Pharmaceutical society	Not stated	Risk of hospital admissions	Not stated	162 medicines stopped of which 40% were high risk, 113 dose/formulation changes and 20 medicines started	162 medications stopped and 113 doses changes	40% of stopped medications were high risk	Not stated

### Outcomes

All sources mention barriers and facilitators to medicines optimisation (Table 6). These include housebound patients’ inability to leave home to receive healthcare (n = 2) [16,17], lack of access to healthcare and reduced mobility increasing medication management challenges of housebound people (n = 1) [37], frail elderly people prescribed medications contributing to falls (n = 1) [36], stroke patients’ inability to visit the pharmacy causing lack of pharmacist contact (n = 1) [35] and vulnerability and polypharmacy of housebound patients (n = 2) [38,39]. Patient satisfaction was reported positively in two papers [17,35], with 100% of patients or carers finding the dMUR helpful in one study [17]. One study found a patient satisfaction rate of 77.8% in the intervention group comparing more favourably that the 76.5% in the usual care group [35]. Only one source allowed decision making to be shared between pharmacists and patients [37]. Shared-decision making was not explicitly described in any papers. Quality of life was discussed in one paper in which two houses were found to have damp, and over 10% of patients with unaddressed mobility problems. [17] The data sources mentioned some methods of healthcare utilisation such as: the role of pharmacists in medicines management (n = 2) [17,37]; prevention of hospital admission (n = 3); [16,38,40] and cost savings (n = 2) [36,38]. No study directly measured the impact of medicines optimisation on hospital admissions. Latif used a pharmacist-applied scoring system to self-assess admission risk and the impact of dMURs: Score 1 = no likelihood; Score 2 = possible; Score 3 = likely emergency hospital admission prevented [16].

**Table 6:** Outcomes.

Author	Barriers and facilitators to medicines optimisation	Patient satisfaction	Shared decision-making	Quality of life	Health care utilization and costs (e.g., hospital admissions, medication costs)	Gaps in literature/ uncertainties identified
Kayyali R, Funnell G, Harrap N, Patel A. 2018	Bringing community pharmacists to housebound patients	133 (100%) patients or carers finding the dMUR helpful	Not stated	Two houses found to have damp and one with major tripping hazards. Over 10% of patients had unaddressed mobility problems two patients had issues preventing them from easily reaching the toilets/bathrooms, leading to poor hygiene.	Community pharmacists may assist in the care pathway to help involve aspects of care of older people with multi-morbidities.	Elements of the patients social networks and connections to assess the best interventions. Access to full medical records would provide a complete understanding of their medical situation. Recordings of the dMURs could allow service improvement.
Garfield SF, Wheeler C, Etkind M, Ogunleye D, Williams M, Boucher C, et al. 2022	Housebound patients may face challenges to their medicines management due to reduced household mobility and potential lack of access to healthcare services.	Not stated	Not stated	Not stated	Pharmacists to support housebound patients by synchronization of medicines, delivering medications and education about the importance of communication	Findings are Uk only and may limit generalisability of findings to other countries. Little work on patient-family perspective of medication needs of housebound patients. Lack of equal research between genders
Hurley D. 2018	Frail elderly patients are often prescribed multiple medications, frequently contributing to falls and hospital admissions. Falls services often overlook medicinal causes	Not stated	Not stated	Not stated	Discontinued medication saving £11,423 (if they were still taking for another 12 months) and interventions preventing 2.51 hospital admissions, saving £7,000 (based on predicted procedures upon admission)	Has never been possible to directly link interventions to prevented hospital admissions
Latif A, Mandane B, Anderson E, Barraclough C, Travis S. 2018	dMURs is typically delivered from a community pharmacy, and patients who are homebound may not be receiving the service.	Not stated	Not stated	Not stated	Role of pharmacists in reducing hospital admissions	Further evaluation needed to assess cost effectiveness. Potential lack of consistency in scoring systems between individual pharmacists. Majority white British population, indicating a lack of patients from underserved communities. No comparison between usual MUR service and dMUR.
Souter C, Kinnear A, Kinnear M, Mead G. 2017	A proportion of patients with stroke on discharge do not have contact with the community pharmacist because they are unable to visit the pharmacy in person.3	77.8% in intervention group 76.5 in usual care	Not stated	Not stated	Not stated	Further feasibility testing for the measurement of BP. Generalisability is difficult due to one researcher visiting all patients, despite this researcher being a clinical pharmacist.
Royal Pharmaceutical society 2014	Housebound patients are often taking a lot of medications	Not stated	Not stated	Not stated	Cost avoidance of £234,000 from reduced hospital admissions	Not stated
Royal Pharmaceutical society	Increased vulnerability of housebound people	Not stated	Not stated	Not stated	Not stated	Not stated

Five sources identified and highlighted gaps and uncertainties within their research as follows: no comparison between standard intervention and research intervention, and lack of patients from underserved communities [16]; investigation of social connections and access to full medical records [17]; difficult to directly link interventions and hospital admissions [36]; unequal gender participation [37]; and lack of generalisability [35].

### Synthesis of results

**Fig 2:**
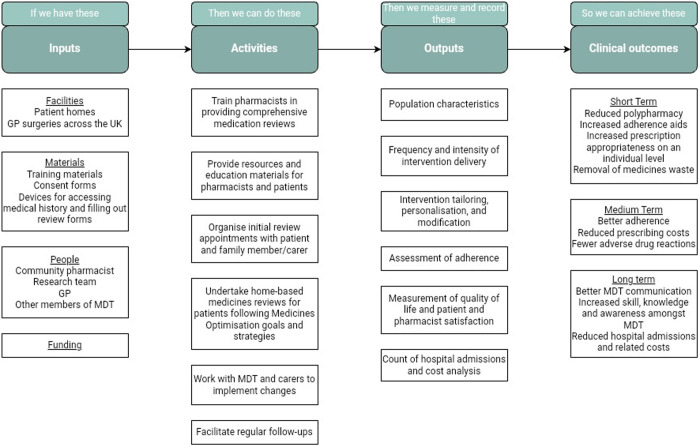
Pipeline logic model of main findings.

Important findings are illustrated in a logic model to summarise the roles community pharmacists may play in medicines optimisation for elderly housebound people (Fig 2).

## Discussion

### Summary of evidence

This scoping review identified a small but growing body of literature on the role of community pharmacists in medicines optimisation for housebound older adults in the UK. The principal findings suggest that pharmacist-led domiciliary medication reviews (dMURs) can effectively identify and address medication-related problems, potentially reducing inappropriate polypharmacy, non-adherence, adverse drug reactions, and hospital admissions in this vulnerable population. None of the included studies assessed the impact of medicines optimisation on hospital admissions using direct measures.

It is important to note that none of the included studies presented findings that contradicted the potential benefits of pharmacist-led interventions for housebound older adults. All included sources either reported positive outcomes or were neutral in their conclusions. The absence of contradictory evidence does not equate to conclusive proof of effectiveness; however, it does support the rationale for further investigation through more rigorous and controlled studies.

### Strengths and limitations

This scoping review involved an extensive search of both academic and grey literature to understand the breadth of evidence in the relevant field. The comprehensive eligibility criteria provided a thorough overview of the topic, permitting consideration of various intervention types, outcomes, and study designs, and providing information on the range of roles available for pharmacists within home-based medicines optimisation. The screening process involved a team of five reviewers to increase austerity.

Established frameworks such as TIDieR and EPOC taxonomy were utilised to ensure data was systematically extracted and narrated, including placing an emphasis on identifying gaps in research, and generating recommendations for future research and practice.

However, this scoping review was intended as a descriptive narrative of available literature, and thus a formal quality appraisal was not conducted to assess the strength of evidence within individual studies. Instead, where there was confusion about the validity of a study, another researcher provided an opinion. Similarly, the grey literature screened for eligibility may not provide commentary on all unpublished case studies, and thus this review may be at risk of publication bias.

The data sources included in this review were restricted to publishing dates after 2000, published in English and relevant to the United Kingdom. This made some relevant literature ineligible for inclusion in the review, and a wider search may be warranted for further review. The methodology of a scoping review does not allow for synthesis of effectiveness data or analysis of methodological limitations of data provided, and therefore a systematic review may be in order.

### Relation to other studies

The findings of this scoping review are broadly consistent with previous studies about pharmacist-led interventions in similar populations. Abbott et al. [41] found no effect on hospital admissions among individuals at risk of medication-related problems receiving pharmacist home visits, while Spinewine et al. [42], reported improvements in pharmacotherapy for older patients. Abbott et al. proposed that a lack of interprofessional communication may have explained the absence of any effect on admissions observed in their systematic review [41]. Specifically, they noted that in the one study showing reduced admissions, the pharmacist routinely communicated findings to both the general practitioner and local pharmacist, a practice rarely reported in other studies [43]. They also suggest that pharmacists conducting home visits alongside normal duties, rather than as dedicated roles, and longer follow-up periods may dilute observable effects [41].

Our review similarly identified mixed evidence on the impact of pharmacist-led medicines optimisation on healthcare utilisation and clinical outcomes for housebound older adults.

The themes identified in our review, such as collaborative working, patient involvement in goal setting and action planning, and the provision of additional support and follow-up, align with the findings of Craske et al. [44], who explored the components of pharmacist-led medication reviews. However, our review extends

these insights by focusing specifically on the unique needs and challenges of housebound older adults and the role of community pharmacists in this context.

Consistent with the conclusions of Saeed et al. [45], who investigated medicines optimization interventions for frail older inpatients, our review found that while pharmacist-led interventions may improve prescribing appropriateness, there is a lack of high-quality evidence on their impact on clinical outcomes in housebound populations.

Our findings also resonate with studies highlighting the importance of pharmacists’ willingness and competence in driving medication optimisation in care home settings [46]. However, the specific focus of our review on housebound older adults in the community setting distinguishes it from research in institutional contexts, where medication management processes and challenges may differ.

### Policy implications

Our findings reinforce NHS England’s guidance that housebound individuals with problematic polypharmacy, frailty, recent hospitalisations or fall risk are key candidates for structured medication reviews and demonstrate the feasibility of pharmacist-led interventions in identifying and addressing such risks in this population [47]. However, the variability in service provision and underrepresentation of underserved groups point to the need for strengthened policy mechanisms to ensure equitable, systematic implementation of structured medication reviews across primary care networks, supported by targeted workforce planning, commissioning frameworks and integration into routine care through formal referral pathways and shared clinical records.

### Future research

While randomised controlled trials are needed to robustly evaluate the effectiveness and cost-effectiveness of pharmacist-led medicines optimisation interventions for housebound older adults, important foundational work is first required. Building on the findings of this scoping review, future research should focus on the co-design of potential interventions in collaboration with key stakeholders, including patients, carers, pharmacists, and other healthcare professionals, to ensure their feasibility, acceptability, and relevance to the specific needs of this population. Frameworks for intervention development [48,49] should be used to guide this process, to integrate diverse perspectives, prioritising intervention components and outcome measures. This developmental work would lay the groundwork for future pilot and definitive evaluations.

## Conclusions

There is a clear need to establish a precedent for caring for this vulnerable population. This scoping review lays the groundwork to build upon existing research in this field, yet significant gaps remain. Understanding the evidence surrounding community pharmacists’ contribution is crucial for developing services that enhance care, reduce adverse events, and promote health equity.

The NHS’ medicines optimisation opportunities [4] indicated a place for pharmacists in providing for this population, and this review highlights the capacity of their role. Given the limited depth of available data, a more systematic approach may be needed to assess the feasibility and impact of specific interventions. With further research, there is a vast opportunity for filling this gap in care.

## Supporting information

S1 Protocol Scoping review protocol. (DOCX)

S2 Checklist PRISMA-ScR Checklist. (DOCX)

S3 Appendix Inclusion and exclusion criteria. (DOCX)

S4 Appendix Search strategy. (DOCX)

S5 Appendix Websites searched for grey literature. (DOCX)

S6 Appendix Data charting process. (DOCX)
