# Supplementary material for: The role of community pharmacists in medicines optimisation for housebound people: A scoping review
Source: PLoS One. 2025 Sep 11;20(9):e0331294. doi: 10.1371/journal.pone.0331294 (PMC12425228; doi:10.1371/journal.pone.0331294)
Supplement: S1 Protocol — (DOCX) [file pone.0331294.s001.docx]

**Title: The Role of Community Pharmacists in Medicines Optimisation for Housebound People: scoping review protocol (Version 1, 1st September 2024)**

Contact

Greg Westley

greg.westley@nhs.net

Daniel Hind

d.hind@sheffield.ac.uk

Contributions of Protocol Authors

Greg Westley and Daniel Hind conceived the review. DH, GW and Richard Cooper designed the review. DH and GW gave final approval for this version to be published.

Guarantor of the review

DH: d.hind@sheffield.ac.uk

**ABSTRACT**

**Introduction:** Housebound older adults in the UK face significant challenges related to medication management, including polypharmacy, inappropriate prescribing, and adverse drug events. Community pharmacists are well-positioned to optimize medicines use in this population, but their specific role remains unclear.

**Methods:** This scoping review will follow PRISMA-ScR guidelines. We will search MEDLINE, Embase, Scopus, Web of Science, grey literature sources, and trial registries for studies on community pharmacists' involvement in medicines optimization for housebound older adults in the UK. Inclusion criteria encompass interventions, services, or initiatives related to medicines optimization involving community pharmacists. Two reviewers will independently screen citations, chart data, and synthesize findings narratively. Formal quality appraisal will not be conducted. Evidence gaps and future research recommendations will be highlighted.

**Background and rationale**

Housebound older adults in the UK face significant challenges related to medication management, which can lead to various adverse outcomes[[1]](https://paperpile.com/c/fSPKRZ/rIxq). These individuals often suffer from multiple chronic conditions requiring complex medication regimens, resulting in a high prevalence of polypharmacy[[1,2]](https://paperpile.com/c/fSPKRZ/1AK5+rIxq). Polypharmacy increases the risk of medication errors, adverse drug reactions, and drug interactions[[1,3,4]](https://paperpile.com/c/fSPKRZ/g0iP+rIxq+EVYn). Studies have shown a significant increase in the number of older adults experiencing polypharmacy internationally, with many of these individuals being at high risk for adverse outcomes[[5]](https://paperpile.com/c/fSPKRZ/SC5G). Economically, medication-related problems contribute significantly to healthcare costs[[5]](https://paperpile.com/c/fSPKRZ/SC5G), with medication errors costing the NHS up to £2.5 billion annually, encompassing various costs such as hospital admissions and additional treatments required to manage the consequences of these errors​ [[6]](https://paperpile.com/c/fSPKRZ/RnGV). The isolation and reduced access to healthcare services further exacerbate these issues[[5]](https://paperpile.com/c/fSPKRZ/SC5G), making effective medication management a critical concern for this vulnerable population.

Polypharmacy, defined as the concurrent use of multiple medications by one individual, has become increasingly common and presents significant challenges for medication management and patient safety[[7–10]](https://paperpile.com/c/fSPKRZ/BLH2+9Kzv+lSq0+2mJI). Between 2003 and 2013, the average number of prescription items per year for any one person in England increased from 13 to 19[[7,11]](https://paperpile.com/c/fSPKRZ/PMtA+BLH2). This trend is particularly pronounced in older adults, with more than 1 in 10 people aged over 65 taking at least eight different prescribed medications each week, increasing to nearly 1 in 4 people aged over 85[[9]](https://paperpile.com/c/fSPKRZ/lSq0).

The prevalence of polypharmacy has risen substantially in recent years. A large Scottish study found that the proportion of patients dispensed 5 or more drugs increased from 12% to 22% between 1995 and 2010, while those dispensed 10 or more drugs increased from 1.9% to 5.8%[[7]](https://paperpile.com/c/fSPKRZ/BLH2). In England, recent data shows that approximately 8.4 million people are taking five or more medicines, and 3.8 million are taking eight or more unique medicines[[9,10]](https://paperpile.com/c/fSPKRZ/2mJI+lSq0). This rise in polypharmacy is driven by an aging population, increasing prevalence of multimorbidity, and a growing tendency to prescribe preventive medications to asymptomatic individuals[[7,9,10]](https://paperpile.com/c/fSPKRZ/2mJI+BLH2+lSq0).

Polypharmacy presents both benefits and risks. While appropriate polypharmacy can improve outcomes for patients with multiple conditions, problematic polypharmacy increases the risk of adverse drug reactions, impairs medication adherence, and can negatively impact quality of life[[7,9,10]](https://paperpile.com/c/fSPKRZ/2mJI+lSq0+BLH2). It is estimated that around 1 in 5 prescriptions for older people living at home may be inappropriate. Adverse reactions to medicines are implicated in up to 6.5% of hospital admissions[[7]](https://paperpile.com/c/fSPKRZ/BLH2) , with a 53% increase in emergency admissions due to adverse drug reactions between 2008 and 2015[[9]](https://paperpile.com/c/fSPKRZ/lSq0).

The economic impact of inappropriate polypharmacy is substantial. In 2017/18, the NHS spent £18.2 billion on prescription medicines, 40% more than in 2010/11[[9]](https://paperpile.com/c/fSPKRZ/lSq0). It is estimated that £300 million of NHS medicines are wasted every year, with up to 50% of all medicines for long-term conditions not taken as intended[[9]](https://paperpile.com/c/fSPKRZ/lSq0). Furthermore, the production and use of medicines can generate significant greenhouse gas emissions, contributing to climate change[[10]](https://paperpile.com/c/fSPKRZ/2mJI).

The concept of medicines optimisation has emerged to address these challenges. Defined by NICE as "a person-centred approach to safe and effective medicines use, to ensure people obtain the best possible outcomes from their medicines," medicines optimisation goes beyond simple medication review[[11]](https://paperpile.com/c/fSPKRZ/PMtA). It encompasses a range of interventions including medicines reconciliation, self-management plans, patient decision aids, and cross-sector collaboration[[11]](https://paperpile.com/c/fSPKRZ/PMtA).

Recent UK studies by Latif et al. and Kayyali et al. have pioneered innovative approaches to medicines review for housebound older adults. These domiciliary Medicines Use Review MUR services represent important steps towards more patient-centered care by reaching a vulnerable population often overlooked by traditional pharmacy services. Both studies demonstrated that pharmacists could identify and address medication-related problems in patients' homes, potentially preventing hospital admissions and improving medication use. However, while valuable, these initiatives fall short of the comprehensive medicines optimisation approach proposed by NICE[[12]](https://paperpile.com/c/fSPKRZ/uMwz). They primarily focus on medication-related issues such as adherence, side effects, and storage, without fully integrating broader health and social care needs or involving multidisciplinary collaboration. A true medicines optimisation approach might go further by considering the patient's overall health goals, involving other healthcare professionals, and addressing non-pharmacological interventions alongside medication management.

Community pharmacists are well-positioned to play a key role in medicines optimisation, particularly for older adults who may have complex medication regimens. The NHS Long Term Plan recognizes this challenge and commits to expanding access to high-quality medicines reviews and ensuring pharmacists are part of local community health teams[[9,10]](https://paperpile.com/c/fSPKRZ/2mJI+lSq0). This includes funding the deployment and specialist training of thousands of clinical pharmacists and pharmacy technicians in Primary Care Networks by 2024[[10]](https://paperpile.com/c/fSPKRZ/2mJI).

However, the specific role of community pharmacists in medicines optimisation for housebound older adults in the UK remains unclear. A scoping review is warranted to map the existing literature on this topic and identify gaps in current knowledge, particularly in light of the growing emphasis on addressing inappropriate polypharmacy and improving medicines management for older adults. This review should also consider the impact of recent initiatives such as Structured Medication Reviews, the NHS Discharge Medicines Service, and increased focus on shared decision-making and personalised care[[10]](https://paperpile.com/c/fSPKRZ/2mJI), while exploring how these services can move beyond medication review to true medicines optimisation for this vulnerable population.

**Objectives:**

We will conduct a scoping review to map the existing literature on the role of community pharmacists in medicines optimisation for housebound older adults in the UK. The review questions will be conceptualized using the PCC (Population, Concept, Context) framework as follows:

- Population: Housebound older adults in the UK

- Concept: The role of community pharmacists in medicines optimisation

- Context: UK community pharmacy setting

**Protocol and registration:**

We have drafted this protocol using the PRISMA Extension for Scoping Reviews (PRISMA-ScR). The review will be registered prospectively with the Open Science Framework (<https://osf.io>) and the protocol published on ORDA. Any amendments made to the protocol will be documented and justified.

**Inclusion criteria:**

Population:

- Housebound older adults (aged 65 years and over) living in domiciliary settings in the UK

- Community pharmacists involved in the care of housebound older adults.

Concept:

- Interventions, services or initiatives related to medicines optimisation that involve community pharmacists, including but not limited to:

- Medication review

- Deprescribing

- Adherence support

- Patient education and counseling

- Addressing polypharmacy and inappropriate prescribing

- Facilitating communication between healthcare providers

- Use of technology or information sharing to support medicines optimisation

Context:

- Community pharmacy services delivered in domiciliary settings in the UK

- Services targeting medicines optimisation for housebound older adults

Types of sources:

- Peer-reviewed primary research studies (quantitative, qualitative or mixed methods)

- Protocols for planned or ongoing studies

- Published conference abstracts with sufficient detail on methods and results

- Grey literature that is one of the following[[13]](https://paperpile.com/c/fSPKRZ/r9EJ):

- Annual reports, business press, case studies, commercial organizations reports, commissioned reports, community engagement toolkits, conference proceedings/papers, consultancy reports, discussion papers, economic impact studies, national and local government departments reports, government reports, industry reports, institutional reports, international organization industry reports, NGO reports, patient opinions, national and local (e.g. NHS or local authority) policy documents, policy-maker consultations, professional guidelines or policy statements, practitioner articles, papers and reports, research reports, teaching cases, Masters and PhD theses, think-tank reports, websites and working papers (Category A)

Exclusion criteria:

Population:

- Studies involving only older adults who are not housebound

- Studies involving only younger adults (<65 years)

- Studies involving only older adults in care homes, hospitals or non-domiciliary settings

Concept:

- Studies of medicines optimisation interventions that do not involve community pharmacists

- Studies focused solely on perspectives of stakeholders other than patients or healthcare professionals

Context:

- Studies conducted outside of the UK

- Studies in settings other than domiciliary care (e.g. care homes, hospitals)

Types of sources:

- Secondary research (e.g. systematic and other reviews, editorials, commentaries, although the references lists of such sources will be screened for eligible articles)

- Grey literature that is one of the following[[13]](https://paperpile.com/c/fSPKRZ/r9EJ):

- newspaper/magazine articles, business press reports, anonymous publications, articles from popular rather than academic sources, book chapters, book reviews, books, commentaries practitioner accounts, undergraduate dissertations, editorials, essays, letters, journalistic or anecdotal articles, literature reviews, monographs, news items, non-refereed publications, opinion pieces, prescriptive accounts, special issue introductory pieces, trade and popular press, unpublished papers, unpublished reports (Category B) Blogs, executive summaries of papers, newspaper articles, PowerPoint files, press releases, reports on the results of public consultations, reports or literature that describe implementation of consultation activities, short practitioner articles, unpublished posters, unsupported prescriptions and webmedia (Category A)

Other restrictions:

- Studies published before the year 2000

- Studies published in languages other than English

**Information sources:**

We will search the following electronic databases from 2000 to the date of search: MEDLINE, Embase. The search will be supplemented by scanning the reference lists of included studies and relevant reviews. Additionally, we will conduct a targeted search for grey literature on websites of relevant organisations and stakeholders. The final search will be conducted in September 2024.

Thank you for the feedback. I have updated the information sources section to include more details on grey literature searching and the additional sources you mentioned:

Information sources:

The following electronic bibliographic databases will be searched: Ovid MEDLINE, Embase, Scopus and Web of Science.

To identify grey literature, the following sources will be searched:

- OpenGrey

- Overton

- The King's Fund

- Nuffield Trust

- The Health Foundation

- NICE Evidence Search

- Royal Pharmaceutical Society

- Department of Health and Social Care

- Age UK

- NIHR Journals Library

- ProQuest Dissertations & Theses Global

Supplementary search methods will include:

- Citation searching of included studies and relevant reviews using Web of Science

- Checking reference lists of included studies and relevant reviews

- Searching Connected Papers for additional relevant publications

Trial registries will also be searched to identify any ongoing or recently completed trials:

- ClinicalTrials.gov

- ISRCTN Registry

- EU Clinical Trials Register

The search will be limited to studies published in English from the year 2000 onwards. The initial database search will be supplemented with weekly alerts to identify any new literature published between the initial search and the completion of the review.

The search process will be reported using a PRISMA flow diagram. The final search strategies for each database and grey literature source will be provided in full in the review appendices.

**Search:**

The full search strategy for MEDLINE is as follows:

1. exp Homebound Persons/

2. (homebound or home-bound or "home bound" or housebound or house-bound or "house bound" or bedbound or bed-bound or "bed bound" or bedfast or bedridden or bed-ridden).ti,ab,kf.

3. House Calls/

4. ("home visit*" or "house call*" or HBPC).ti,ab,kf.

5. ((home or house) adj (based or bound)).ti,ab,kf.

6. (home adj2 (care or healthcare or service* or visit*)).ti,ab,kf.

7. 1 or 2 or 3 or 4 or 5 or 6

8. exp Aged/ or exp "Aged, 80 and over"/

9. (aged or elder* or older or geriatric* or senior* or "old people" or "old person*").ti,ab,kf.

10. 8 or 9

11. exp Medication Therapy Management/

12. "Drug Utilization Review"/

13. Medication Reconciliation/

14. Inappropriate Prescribing/

15. Medication Adherence/

16. Deprescriptions/

17. ((medication* or medicine* or drug*) adj2 (management or optimi?ation or reconciliation or review* or utili?ation or regime)).ti,ab,kf.

18. ((medication* or medicine* or prescrib*) adj2 (appropriate* or inappropriate* or over or under or error*)).ti,ab,kf.

19. (deprescrib* or deprescription* or "de prescrib*" or "de prescription*").ti,ab,kf.

20. ((medication* or medicine*) adj2 (adherence or compliance or non-adherence or noncompliance or persist*)).ti,ab,kf.

21. ((improv* or optimi?*) adj2 (prescrib* or prescription*)).ti,ab,kf.

22. (pharmaceutical adj2 care).ti,ab,kf.

23. 11 or 12 or 13 or 14 or 15 or 16 or 17 or 18 or 19 or 20 or 21 or 22

24. exp Great Britain/

25. (national health service* or NHS*).ti,ab,in.

26. (english not ((published or publication* or translat* or written or language* or speak* or literature or citation*) adj5 english)).ti,ab.

27. (gb or "g.b." or britain* or (british* not "british columbia") or uk or "u.k." or united kingdom* or (england* not "new england") or northern ireland* or northern irish* or scotland* or scottish* or ((wales or "south wales") not "new south wales") or welsh*).ti,ab,in,jw.

28. (bath or "bath's" or ((birmingham not alabama*) or ("birmingham's" not alabama*) or bradford or "bradford's" or brighton or "brighton's" or bristol or "bristol's" or carlisle* or "carlisle's" or (cambridge not (massachusetts* or boston* or harvard*)) or ("cambridge's" not (massachusetts* or boston* or harvard*)) or (canterbury not zealand*) or ("canterbury's" not zealand*) or chelmsford or "chelmsford's" or chester or "chester's" or chichester or "chichester's" or coventry or "coventry's" or derby or "derby's" or (durham not (carolina* or nc)) or ("durham's" not (carolina* or nc)) or ely or "ely's" or exeter or "exeter's" or gloucester or "gloucester's" or hereford or "hereford's" or hull or "hull's" or lancaster or "lancaster's" or leeds* or leicester or "leicester's" or (lincoln not nebraska*) or ("lincoln's" not nebraska*) or (liverpool not (new south wales* or nsw)) or ("liverpool's" not (new south wales* or nsw)) or ((london not (ontario* or ont or toronto*)) or ("london's" not (ontario* or ont or toronto*)) or manchester or "manchester's" or (newcastle not (new south wales* or nsw)) or ("newcastle's" not (new south wales* or nsw)) or norwich or "norwich's" or nottingham or "nottingham's" or oxford or "oxford's" or peterborough or "peterborough's" or plymouth or "plymouth's" or portsmouth or "portsmouth's" or preston or "preston's" or ripon or "ripon's" or salford or "salford's" or salisbury or "salisbury's" or sheffield or "sheffield's" or southampton or "southampton's" or st albans or stoke or "stoke's" or sunderland or "sunderland's" or truro or "truro's" or wakefield or "wakefield's" or wells or westminster or "westminster's" or winchester or "winchester's" or wolverhampton or "wolverhampton's" or (worcester not (massachusetts* or boston* or harvard*)) or ("worcester's" not (massachusetts* or boston* or harvard*)) or (york not ("new york*" or ny or ontario* or ont or toronto*)) or ("york's" not ("new york*" or ny or ontario* or ont or toronto*))))).ti,ab,in.

29. (bangor or "bangor's" or cardiff or "cardiff's" or newport or "newport's" or st asaph or "st asaph's" or st davids or swansea or "swansea's").ti,ab,in.

30. (aberdeen or "aberdeen's" or dundee or "dundee's" or edinburgh or "edinburgh's" or glasgow or "glasgow's" or inverness or (perth not australia*) or ("perth's" not australia*) or stirling or "stirling's").ti,ab,in.

31. (armagh or "armagh's" or belfast or "belfast's" or lisburn or "lisburn's" or londonderry or "londonderry's" or derry or "derry's" or newry or "newry's").ti,ab,in.

32. 24 or 25 or 26 or 27 or 28 or 29 or 30 or 31

33. 7 and 10 and 23 and 32

34. limit 33 to (english language and yr="2000 -Current")

**Selection of sources of evidence:**

The results of the search will be collated and deduplicated using reference management software. Two reviewers will independently screen the titles and abstracts against the eligibility criteria. Potentially relevant full texts will then be obtained and screened independently by two reviewers. Any disagreements will be resolved through discussion or referral to a third reviewer. The selection process will be reported using a PRISMA 2020 flow diagram.

**Data charting process:**

A standardized data charting form will be developed and piloted on a sample of included studies. The form will be modified as needed during the course of the review. Two reviewers will independently chart the data, with any disagreements resolved through discussion or a third reviewer. Missing or unclear information will be sought from study authors. The charting process will be documented in detail to ensure reproducibility.

**Data items:**

We will chart data for the following:

Document characteristics:

- First author

- Year

- Document type (grey literature; peer reviewed article; conference abstract)

- For peer reviewed literature and conference abstracts only, defined in terms of:

- Yazdani’s Taxonomy of Research Studies[[14]](https://paperpile.com/c/fSPKRZ/zk4B) supplemented by:

- Peinemann’s algorithm for describing quantitative studies[[15]](https://paperpile.com/c/fSPKRZ/eBFH).

- For grey literature only, defined using Adams’ typology (see eligibility criteria, above) [[13]](https://paperpile.com/c/fSPKRZ/r9EJ).

Participant characteristics:

- Patient group: Case mix, number of patients, patient demographics (e.g. age, gender, type of cancer, medical conditions, numbers and types of medicines) [Murphy]

TIDieR[[16]](https://paperpile.com/c/fSPKRZ/ao6n) item 1 Brief name:

- Intervention name or phrase

TIDieR item 2 Why:

- Rationale, evidence-base theory or goal of intervention elements

- Details of the intervention development process (e.g. use of existing evidence base/theory)

TIDieR item 3 What (materials):

- Physical or informational materials used (information materials; training materials)

- Intervention costs/resource requirements

TIDieR item 4 What (procedures):

- Procedures, activities, processes used

- EPOC taxonomy[[17]](https://paperpile.com/c/fSPKRZ/gAxa) Intervention category:

- 'Delivery arrangements'

- 'Financial arrangements'

- 'Governance arrangements'

- 'Implementation strategies')

- Intervention duration

TIDieR item 5 Who provided:

- Intervention provider background/expertise (pharmacist; nurse; physician; multidisciplinary team)

- Number of intervention providers

- Any specific training given to providers

TIDieR item 6 How:

- Modes of delivery (e.g. face-to-face; telephone; Internet)

- Whether delivered individually or in a group

TIDieR item 7 Where:

- Number of locations

- Necessary infrastructure or relevant features of locations

- Organisation: Type, size, number of study sites, ratings

- Location: Area demographics

TIDieR item 8 When and how much:

- Number of times delivered

- Schedule of delivery

- Duration of delivery

- Intensity or dose (drug dose/frequency changes)

TIDieR item 9 Tailoring:

- If intervention was personalised or adapted, how and why (e.g. based on patient characteristics)

TIDieR item 10 Modifications:

- If modified during the study, what changes were made, how and why

TIDieR item 11 How well (planned):

- If/how adherence or fidelity was assessed (e.g. independent assessors; validated tools)

Outcome characteristics: Outcome measures used to evaluate the intervention (reported outcomes, assessment methods and time points)

- Barriers and facilitators to medicines optimisation

- Experiences of pharmacists, patients and other stakeholders

- Outcomes in relation to medicines optimisation:

- Prescribing appropriateness (e.g. number and type of medication changes, use of tools like MAI, Beers criteria)

- Adverse drug reactions

- Adherence

- Patient satisfaction

- Shared decision-making

- Quality of life

- Health care utilization and costs (e.g. hospital admissions, medication costs)

Research gaps:

- Details of any gaps in the existing literature or uncertainties identified

Research Recommendations

**Critical appraisal of individual sources of evidence:**

As this is a scoping review, we will not conduct a formal appraisal of the methodological quality or risk of bias of the included studies. This is consistent with guidance for conducting scoping reviews[[18]](https://paperpile.com/c/fSPKRZ/7Wza).

**Synthesis of results:**

We will provide a narrative synthesis of the charted data in relation to the review questions and objectives. Tables and figures will be used to map the evidence and present a summary of key characteristics of the included studies[[19]](https://paperpile.com/c/fSPKRZ/JWTk). Quantitative findings will be descriptively summarised. Qualitative findings related to barriers, facilitators and experiences will be thematically synthesised. Evidence gaps and recommendations for future research and practice will be highlighted.

**References**

1. [Molokhia M, Majeed A. Current and future perspectives on the management of polypharmacy. BMC Fam Pract. 2017;18: 70.](http://paperpile.com/b/fSPKRZ/rIxq)

2. [Website. Available:](http://paperpile.com/b/fSPKRZ/1AK5) <https://www.kingsfund.org.uk/publications/polypharmacy-and-medicines-optimisation>

3. [Maher RL, Hanlon J, Hajjar ER. Clinical consequences of polypharmacy in elderly. Expert Opin Drug Saf. 2014;13: 57–65.](http://paperpile.com/b/fSPKRZ/g0iP)

4. [Khezrian M, McNeil CJ, Murray AD, Myint PK. An overview of prevalence, determinants and health outcomes of polypharmacy. Ther Adv Drug Saf. 2020;11: 2042098620933741.](http://paperpile.com/b/fSPKRZ/EVYn)

5. [Cook EA, Duenas M, Harris P. Polypharmacy in the Homebound Population. Clin Geriatr Med. 2022;38: 685–692.](http://paperpile.com/b/fSPKRZ/SC5G)

6. [Torjesen I. Medication errors cost the NHS up to £2.5bn a year. In: The Pharmaceutical Journal [Internet]. 20 Oct 2014 [cited 21 Jul 2024]. Available:](http://paperpile.com/b/fSPKRZ/RnGV) <https://pharmaceutical-journal.com/article/news/medication-errors-cost-the-nhs-up-to-2-5bn-a-year>

7. [Duerden M, Avery T, Payne R. Polypharmacy and Medicines Optimisation: Making It Safe and Sound. 2013.](http://paperpile.com/b/fSPKRZ/BLH2)

8. [National Institute for Health and Care Excellence. Medicines optimisation: the safe and effective use of medicines to enable best possible outcomes (NG5). London: NICE; 2015.](http://paperpile.com/b/fSPKRZ/9Kzv)

9. [Are W we. Age UK calls for a more considered approach to prescribing medicines for our older population. In: Age UK [Internet]. [cited 21 Jul 2024]. Available:](http://paperpile.com/b/fSPKRZ/lSq0) <https://www.ageuk.org.uk/latest-press/articles/2019/august/age-uk-calls-for-a-more-considered-approach-to-prescribing-medicines-for-older-people/>

10. [National overprescribing review report. In: GOV.UK [Internet]. 22 Sep 2021 [cited 21 Jul 2024]. Available:](http://paperpile.com/b/fSPKRZ/2mJI) <https://www.gov.uk/government/publications/national-overprescribing-review-report>

11. [Overview | Medicines optimisation: the safe and effective use of medicines to enable the best possible outcomes | Guidance | NICE. [cited 21 Jul 2024]. Available:](http://paperpile.com/b/fSPKRZ/PMtA) <https://www.nice.org.uk/guidance/ng5>

12. [Overview | Medicines optimisation: the safe and effective use of medicines to enable the best possible outcomes | Guidance | NICE. [cited 21 Jul 2024]. Available:](http://paperpile.com/b/fSPKRZ/uMwz) <https://www.nice.org.uk/guidance/ng5>

13. [Adams RJ, Smart P, Huff AS. Shades of grey: Guidelines for working with the grey literature in systematic reviews for management and organizational studies. Int J Manag Rev. 2017;19: 432–454.](http://paperpile.com/b/fSPKRZ/r9EJ)

14. [Yazdani S, Shirvani A, Heidarpoor P. A model for the taxonomy of research studies: A practical guide to knowledge production and knowledge management. Arch Pediatr Infect Dis. 2021;9. doi:](http://paperpile.com/b/fSPKRZ/zk4B)[10.5812/pedinfect.112456](http://dx.doi.org/10.5812/pedinfect.112456)

15. [Peinemann F, Kleijnen J. Development of an algorithm to provide awareness in choosing study designs for inclusion in systematic reviews of healthcare interventions: a method study. BMJ Open. 2015;5: e007540.](http://paperpile.com/b/fSPKRZ/eBFH)

16. [Hoffmann TC, Glasziou PP, Boutron I, Milne R, Perera R, Moher D, et al. Better reporting of interventions: template for intervention description and replication (TIDieR) checklist and guide. BMJ. 2014;348: g1687.](http://paperpile.com/b/fSPKRZ/ao6n)

17. [EPOC Taxonomy. [cited 21 Jul 2024]. Available:](http://paperpile.com/b/fSPKRZ/gAxa) <https://epoc.cochrane.org/epoc-taxonomy>

18. [Khalil H, Tricco AC. Differentiating between mapping reviews and evidence gap maps. Journal of clinical epidemiology. 2023. pp. 154–155.](http://paperpile.com/b/fSPKRZ/7Wza)

19. [South E, Rodgers M. Data visualisation in scoping reviews and evidence maps on health topics: a cross-sectional analysis. Syst Rev. 2023;12: 142.](http://paperpile.com/b/fSPKRZ/JWTk)
