# Supplementary material for: The role of community pharmacists in medicines optimisation for housebound people: A scoping review
Source: PLoS One. 2025 Sep 11;20(9):e0331294. doi: 10.1371/journal.pone.0331294 (PMC12425228; doi:10.1371/journal.pone.0331294)
Supplement: S3 Appendix — (DOCX) [file pone.0331294.s003.docx]

# S3 Appendix

**Inclusion criteria:**

Population:

- Housebound older adults [aged 65 years and over] living in domiciliary settings in the UK

- Community pharmacists involved in the care of housebound older adults.

Concept:

- Interventions, services or initiatives related to medicines optimisation that involve community pharmacists, including but not limited to:

- Medication review

- Deprescribing

- Adherence support

- Patient education and counselling

- Addressing polypharmacy and inappropriate prescribing

- Facilitating communication between healthcare providers

- Use of technology or information sharing to support medicines optimisation

Context:

- Community pharmacy services delivered in domiciliary settings in the UK

- Services targeting medicines optimisation for housebound older adults

Types of sources:

- Peer-reviewed primary research studies [quantitative, qualitative or mixed methods]

- Protocols for planned or ongoing studies

- Published conference abstracts with sufficient detail on methods and results

- Grey literature that is one of the following:

- Annual reports, business press, case studies, commercial organizations reports, commissioned reports, community engagement toolkits, conference proceedings/papers, consultancy reports, discussion papers, economic impact studies, national and local government departments reports, government reports, industry reports, institutional reports, international organization industry reports, NGO reports, patient opinions, national and local [e.g. NHS or local authority] policy documents, policy-maker consultations, professional guidelines or policy statements, practitioner articles, papers and reports, research reports, teaching cases, Masters and PhD theses, think-tank reports, websites and working papers [Category A]

**Exclusion criteria:**

Population:

- Studies involving only older adults who are not housebound

- Studies involving only younger adults [<65 years]

- Studies involving only older adults in care homes, hospitals or non-domiciliary settings

Concept:

- Studies of medicines optimisation interventions that do not involve community pharmacists

- Studies focused solely on perspectives of stakeholders other than patients or healthcare professionals

Context:

- Studies conducted outside of the UK

- Studies in settings other than domiciliary care [e.g. care homes, hospitals]

Types of sources:

- Secondary research [e.g. systematic and other reviews, editorials, commentaries, although the references lists of such sources will be screened for eligible articles]

- Grey literature that is one of the following:

- newspaper/magazine articles, business press reports, anonymous publications, articles from popular rather than academic sources, book chapters, book reviews, books, commentaries practitioner accounts, undergraduate dissertations, editorials, essays, letters, journalistic or anecdotal articles, literature reviews, monographs, news items, non-refereed publications, opinion pieces, prescriptive accounts, special issue introductory pieces, trade and popular press, unpublished papers, unpublished reports [Category B] Blogs, executive summaries of papers, newspaper articles, PowerPoint files, press releases, reports on the results of public consultations, reports or literature that describe implementation of consultation activities, short practitioner articles, unpublished posters, unsupported prescriptions and webmedia [Category A]

Other restrictions:

- Studies published before the year 2000

- Studies published in languages other than English
