# Supplementary material for: The role of community pharmacists in medicines optimisation for housebound people: A scoping review
Source: PLoS One. 2025 Sep 11;20(9):e0331294. doi: 10.1371/journal.pone.0331294 (PMC12425228; doi:10.1371/journal.pone.0331294)
Supplement: S4 Appendix — (DOCX) [file pone.0331294.s004.docx]

## S4 Appendix

The full search strategy for this review is as below:

1. exp Homebound Persons/

2. [homebound or home-bound or "home bound" or housebound or house-bound or "house bound" or bedbound or bed-bound or "bed bound" or bedfast or bedridden or bed-ridden].ti,ab,kf.

3. House Calls/

4. ["home visit*" or "house call*" or HBPC].ti,ab,kf.

5. [[home or house] adj [based or bound]].ti,ab,kf.

6. [home adj2 [care or healthcare or service* or visit*]].ti,ab,kf.

7. 1 or 2 or 3 or 4 or 5 or 6

8. exp Aged/ or exp "Aged, 80 and over"/

9. [aged or elder* or older or geriatric* or senior* or "old people" or "old person*"].ti,ab,kf.

10. 8 or 9

11. exp Medication Therapy Management/

12. "Drug Utilization Review"/

13. Medication Reconciliation/

14. Inappropriate Prescribing/

15. Medication Adherence/

16. Deprescriptions/

17. [[medication* or medicine* or drug*] adj2 [management or optimi?ation or reconciliation or review* or utili?ation or regime]].ti,ab,kf.

18. [[medication* or medicine* or prescrib*] adj2 [appropriate* or inappropriate* or over or under or error*]].ti,ab,kf.

19. [deprescrib* or deprescription* or "de prescrib*" or "de prescription*"].ti,ab,kf.

20. [[medication* or medicine*] adj2 [adherence or compliance or non-adherence or noncompliance or persist*]].ti,ab,kf.

21. [[improv* or optimi?*] adj2 [prescrib* or prescription*]].ti,ab,kf.

22. [pharmaceutical adj2 care].ti,ab,kf.

23. 11 or 12 or 13 or 14 or 15 or 16 or 17 or 18 or 19 or 20 or 21 or 22

24. exp Great Britain/

25. [national health service* or NHS*].ti,ab,in.

26. [english not [[published or publication* or translat* or written or language* or speak* or literature or citation*] adj5 english]].ti,ab.

27. [gb or "g.b." or britain* or [british* not "british columbia"] or uk or "u.k." or united kingdom* or [england* not "new england"] or northern ireland* or northern irish* or scotland* or scottish* or [[wales or "south wales"] not "new south wales"] or welsh*].ti,ab,in,jw.

28. [bath or "bath's" or [[birmingham not alabama*] or ["birmingham's" not alabama*] or bradford or "bradford's" or brighton or "brighton's" or bristol or "bristol's" or carlisle* or "carlisle's" or [cambridge not [massachusetts* or boston* or harvard*]] or ["cambridge's" not [massachusetts* or boston* or harvard*]] or [canterbury not zealand*] or ["canterbury's" not zealand*] or chelmsford or "chelmsford's" or chester or "chester's" or chichester or "chichester's" or coventry or "coventry's" or derby or "derby's" or [durham not [carolina* or nc]] or ["durham's" not [carolina* or nc]] or ely or "ely's" or exeter or "exeter's" or gloucester or "gloucester's" or hereford or "hereford's" or hull or "hull's" or lancaster or "lancaster's" or leeds* or leicester or "leicester's" or [lincoln not nebraska*] or ["lincoln's" not nebraska*] or [liverpool not [new south wales* or nsw]] or ["liverpool's" not [new south wales* or nsw]] or [[london not [ontario* or ont or toronto*]] or ["london's" not [ontario* or ont or toronto*]] or manchester or "manchester's" or [newcastle not [new south wales* or nsw]] or ["newcastle's" not [new south wales* or nsw]] or norwich or "norwich's" or nottingham or "nottingham's" or oxford or "oxford's" or peterborough or "peterborough's" or plymouth or "plymouth's" or portsmouth or "portsmouth's" or preston or "preston's" or ripon or "ripon's" or salford or "salford's" or salisbury or "salisbury's" or sheffield or "sheffield's" or southampton or "southampton's" or st albans or stoke or "stoke's" or sunderland or "sunderland's" or truro or "truro's" or wakefield or "wakefield's" or wells or westminster or "westminster's" or winchester or "winchester's" or wolverhampton or "wolverhampton's" or [worcester not [massachusetts* or boston* or harvard*]] or ["worcester's" not [massachusetts* or boston* or harvard*]] or [york not ["new york*" or ny or ontario* or ont or toronto*]] or ["york's" not ["new york*" or ny or ontario* or ont or toronto*]]]]].ti,ab,in.

29. [bangor or "bangor's" or cardiff or "cardiff's" or newport or "newport's" or st asaph or "st asaph's" or st davids or swansea or "swansea's"].ti,ab,in.

30. [aberdeen or "aberdeen's" or dundee or "dundee's" or edinburgh or "edinburgh's" or glasgow or "glasgow's" or inverness or [perth not australia*] or ["perth's" not australia*] or stirling or "stirling's"].ti,ab,in.

31. [armagh or "armagh's" or belfast or "belfast's" or lisburn or "lisburn's" or londonderry or "londonderry's" or derry or "derry's" or newry or "newry's"].ti,ab,in.

32. 24 or 25 or 26 or 27 or 28 or 29 or 30 or 31

33. 7 and 10 and 23 and 32

34. limit 33 to [english language and yr="2000 -Current"]
