# Supplementary material for: The role of community pharmacists in medicines optimisation for housebound people: A scoping review
Source: PLoS One. 2025 Sep 11;20(9):e0331294. doi: 10.1371/journal.pone.0331294 (PMC12425228; doi:10.1371/journal.pone.0331294)
Supplement: S5 Appendix — (DOCX) [file pone.0331294.s005.docx]

# S5 Appendix

- Overton
- The King's Fund
- Nuffield Trust
- The Health Foundation
- NICE Evidence Search
- Royal Pharmaceutical Society
- Department of Health and Social Care
- Age UK
- NIHR Journals Library
- ProQuest Dissertations & Theses Global
