# Supplementary material for: The role of community pharmacists in medicines optimisation for housebound people: A scoping review
Source: PLoS One. 2025 Sep 11;20(9):e0331294. doi: 10.1371/journal.pone.0331294 (PMC12425228; doi:10.1371/journal.pone.0331294)
Supplement: S6 Appendix — (DOCX) [file pone.0331294.s006.docx]

# S6 Appendix

The full list of data charted for eligible papers during this review is as follows:

Document characteristics:

- First author

- Year

- Document type (grey literature; peer reviewed article; conference abstract)

Participant characteristics:

- Patient group: Case mix, number of patients, patient demographics (e.g. age, gender, type of cancer, medical conditions, numbers and types of medicines)

TIDieR item 1 Brief name:

- Intervention name or phrase

TIDieR item 2 Why:

- Rationale, evidence-base theory or goal of intervention elements

- Details of the intervention development process (e.g. use of existing evidence base/theory)

TIDieR item 3 What (materials):

- Physical or informational materials used (information materials; training materials)

- Intervention costs/resource requirements

TIDieR item 4 What (procedures):

- Procedures, activities, processes used

    - EPOC taxonomy Intervention category:

              - 'Delivery arrangements'

              - 'Financial arrangements'

              - 'Governance arrangements'

              - 'Implementation strategies')

- Intervention duration

TIDieR item 5 Who provided:

- Intervention provider background/expertise (pharmacist; nurse; physician; multidisciplinary team)

- Number of intervention providers

- Any specific training given to providers

TIDieR item 6 How:

- Modes of delivery (e.g. face-to-face; telephone; Internet)

- Whether delivered individually or in a group

TIDieR item 7 Where:

- Number of locations

- Necessary infrastructure or relevant features of locations

- Organisation: Type, size, number of study sites, ratings

- Location: Area demographics

TIDieR item 8 When and how much:

- Number of times delivered

- Schedule of delivery

- Duration of delivery

- Intensity or dose (drug dose/frequency changes)

TIDieR item 9 Tailoring:

- If intervention was personalised or adapted, how and why (e.g. based on patient characteristics)

TIDieR item 10 Modifications:

- If modified during the study, what changes were made, how and why

TIDieR item 11 How well (planned):

- If/how adherence or fidelity was assessed (e.g. independent assessors; validated tools)

Outcome characteristics: Outcome measures used to evaluate the intervention (reported outcomes, assessment methods and time points)

- Barriers and facilitators to medicines optimisation

- Experiences of pharmacists, patients and other stakeholders

- Outcomes in relation to medicines optimisation:

    - Prescribing appropriateness

    - Adverse drug reactions

    - Adherence

    - Patient satisfaction

    - Shared decision-making

    - Quality of life

    - Health care utilisation and costs (e.g. hospital admissions, medication costs)

Research gaps:

- Details of any gaps in the existing literature or uncertainties identified

Research Recommendations
